# Management of vaccine-related issues during a pandemic emergency: activation of a referral center: Christian Cintori

**DOI:** 10.1093/eurpub/ckac131.077

**Published:** 2022-10-25

**Authors:** C Cintori, G Diegoli, G Mattei, G Belloli, P Viale, L Attard, L Marconi, C Lugli, D Azzalini, C Artoni

**Affiliations:** 1 Regional Health Authority, Emilia-Romagna Region, Bologna, Italy; 2 Infectious Diseases Unit, IRCCS University Hospital of Bologna, Policlinico Sant'Orsola, Bologna, Italy; 3 Department of Medical and Surgical Sciences, Alma Mater Studiorum University of Bologna, Bologna, Italy; 4 School of Hygiene and Preventive Medicine, University of Modena and Reggio Emilia, Modena, Italy; 5 School of Hygiene and Preventive Medicine, University of Ferrara, Ferrara, Italy

## Abstract

**Issue:**

Vaccine hesitancy (VH) and the challenges faced by healthcare workers (HWs) in evaluating the complex risk-benefit ratio of vaccines’ threaten the effectiveness of vaccination policy. The threat is enhanced when new vaccines are adopted during a pandemic emergency. In Italy, the Emilia-Romagna Region (ERR) created a specialized referral board called Vax-Consilium (VC) to support and guide HWs.

**Description of the problem:**

During a pandemic emergency, rapid and appropriate vaccine implementation is necessary to protect fragile individuals and to encourage vaccine adherence among exposed groups. Challenges in the realm of vaccination emerge, especially when dealing with patients with a complex medical history or previous vaccine adverse events. HWs were able to consult VC via a standardized digital form after obtaining the patient’s informed consent. After a multidisciplinary and evidence-based evaluation, VC provided a conclusive report on the individual vaccine risk-benefit analysis. No cost is charged to the patient.

**Results:**

During the anti-COVID-19 vaccination campaign in 2021, 148 interrogations were submitted to VC: 121 were evaluated, whereas 27 were withdrawn by the HWs or rejected because of insufficient documentation. Mean patient age was 44 years. No absolute contraindication was found, whereas in 23 cases VC recommended immunization with a different vaccine. The disciplines most frequently involved were neurology, angiology and cardiology.

**Lessons:**

VC implementation in EER proved highly effective. Indeed, during the pandemic, anti-COVID-19 vaccination coverage reached >90%. In addition, DTaP-polio-HBV-HIb and MMR vaccination coverage reached >95%. VC proved to be a high-quality public health service. Not only was citizens’ trust in the healthcare system enhanced and was VH reduced, but HWs knowledge improved even in cases not considered in national and international guidelines.

**Key messages:**

• A specialized referral board (Vax-Consilium) could be an effective tool for enhancing citizens’ trust in vaccines.

• A specialized referral board (Vax-Consilium) contributes to lowering VH and supporting HWs decision-making process.

